# The effect of sputum quality and volume on the yield of bacteriologically-confirmed TB by Xpert MTB/RIF and smear

**DOI:** 10.11604/pamj.2019.33.110.15319

**Published:** 2019-06-13

**Authors:** Onani Zimba, Tsaone Tamuhla, Joyce Basotli, Gaoraelwe Letsibogo, Sherri Pals, Unami Mathebula, Anikie Mathoma, Christopher Serumola, Kitso Ramogale, Rosanna Boyd, Tiffany Tran, Alyssa Finlay, Andrew Auld, Anand Date, Heather Alexander, Violet Chihota, Tefera Agizew

**Affiliations:** 1 Centers for Disease Control and Prevention, Botswana; 2 Botswana University of Pennsylvania, Botswana; 3 National Tuberculosis Reference Laboratory, Ministry of Health and Wellness, Botswana; 4 Division of Global HIV and Tuberculosis, Centers for Disease Control and Prevention, Atlanta, Georgia, United States of America; 5 Division of Tuberculosis Elimination, Centers for Disease Control and Prevention, Atlanta Georgia, United States of America; 6 The Aurum Institute, Johannesburg, South Africa; 7 School of Public Health, Faculty of Health Sciences, University of Witwatersrand, Johannesburg, South Africa; 8 Department of Family Medicine and Public Health, Faculty of Medicine, University of Botswana, Botswana

**Keywords:** Molecular diagnostic yield, sputum quality, Botswana

## Abstract

**Introduction:**

The World Health Organization endorsed (2010) the use of Xpert MTB/RIF and countries are shifting from smear microscopy (smear)-based to Xpert MTB/RIF-based tuberculosis (TB) diagnostic algorithms. As with smear, sputum quality may predict the likelihood of obtaining a bacteriologically-confirmed TB when using Xpert MTB/RIF.

**Methods:**

From 08/12-11/2014, all people living with HIV were recruited at 22 clinics. For patients screened positive using the four TB symptoms their sputa were tested by Xpert MTB/RIF and smear. Laboratorians assessed and recorded sputum appearance and volume. The yield of bacteriologically-positive sputum evaluated using Xpert MTB/RIF and smear, likelihood-ratios were calculated.

**Results:**

Among 6,041 patients enrolled 2,296 were presumptive TB, 1,305 (56.8%) had > 1 sputa collected and 644/1,305 (49.3%) had both Xpert MTB/RIF and smear results. Since >1 sputa collected from 644 patients 954 sputa were tested by Xpert MTB/RIF and smear. Bacteriologically-positive sputum was two-fold higher with Xpert MTB/RIF 11.4% versus smear 5.3%, p < 0.001. Sputum appearance and quantity were not predictive of bacteriologically-positive results, except volume of 2ml to < 3ml, tested by Xpert MTB/RIF LR+= 1.26 (95% CI, 1.05–1.50).

**Conclusion:**

Xpert MTB/RIF test yield to bacteriologically-positive sputum was superior to smear. Sputum quality and quantity, however, were not consistently predictive of bacteriologically-positive results by Xpert MTB/RIF or smear.

## Introduction

After endorsement by the World Health Organization (WHO) in 2010, over 145 countries implemented the Xpert MTB/RIF assay by 2016 [1]. With such an increased capacity many countries are shifting from a smear microscopy (smear)-to an Xpert MTB/RIF -based tuberculosis (TB) diagnostic algorithm. In 2011, the Botswana Ministry of Health and Wellness adopted WHO guidelines and incorporated Xpert MTB/RIF into the national TB diagnostic algorithm [2]. Optimal performance of Xpert MTB/RIF relies on the quality of the sputum samples submitted for testing [3]. The WHO quality control standard underscores collection of quality sputum, and salivary samples were considered suboptimal and thus prone to rejection by testing laboratories [4]. The minimum required raw sputum sample for smear is 3 - 5 ml [5] compared to 1ml for Xpert MTB/RIF per the Cepheid manufacturer (Cepheid, Sunnyvale, CA, USA) recommendation [6, 7]. Yoon *et al.* and Bhat *et al.*demonstrated that sputum gross appearance (quality) and volume (quantity) were associated with smear positivity [8, 9]. Similar to smear result, sputum quality and quantity may have an impact on the likelihood of obtaining a positive result when using Xpert MTB/RIF [3]. While national TB programs are expanding implementation of Xpert MTB/RIF [1], focus on collection of quality sputum with adequate volume has not been given priority [3]. Data on the effect of sputum quality and quantity on the yield of bacteriologically-confirmed TB using molecular tests such as Xpert MTB/RIF are scarce [3, 10]. We evaluated the proportion of bacteriologically-positive sputum samples with Mycobacterium tuberculosis (MTB) detected using Xpert MTB/RIF, compared to acid fast bacilli identified by smear, stratified by sputum quality and volume.

## Methods

### Study population

This is a sub-study of the Xpert MTB/RIF Package Rollout Evaluation Study using a stepped-wedge design (XPRES), registered at ClinicalTrials.gov, NCT02538952. Full details of the study protocol, including study populations, sample size, and procedures can be accessed in the published protocol [11]. XPRES enrolment began in 2012 as part of the Botswana's national Xpert MTB/RIF rollout, together with intensified TB case finding (ICF) activities and strengthening HIV patient retention interventions at 22 HIV treatment clinics prior to phased implementation of 13 GeneXpert instruments.

### Tuberculosis screening

At enrolment and each follow-up visit (i.e., at two weeks, then monthly for the first three months and then quarterly for the remaining follow up period), adults and adolescents (combined into one adult group and defined as persons >12 years) and children (0-12 years old) were screened for TB symptoms. Per protocol, adults were screened for four TB symptoms (cough, fever, night-sweats and weight-loss) of any duration. Children were screened for weight-loss or failure to thrive (no weight gain over 3 months, enlarged lymph nodes (more than 1 x 1 cm), ≥ 2 weeks of cough, fever, fatigue/reduced playfulness, and profuse night-sweats [2, 12].

### Sputum collection, and assessment of macroscopic sputum quality

Patients who screened positive for any of these TB symptoms or signs were requested to provide four sputa; two were provided on the same day (Spot 1 and 2) and another two on the following day (Morning sputum collected at home and Spot 3 collected at the clinic). In the main study, XPRES, after GeneXpert instrument implementation each Spot 1 and 3 sputum specimen was tested by both Xpert MTB/RIF and smear at the peripheral laboratory. The focus of the sub-analysis was on patients who had at least one TB symptom and from whom at least one sputum was collected for both Xpert MTB/RIF and smear testing. Before GeneXpert instrument implementation each Spot 1 and 3 sputum specimen was tested only by smear at the peripheral laboratory as part of the main study. Spot 2 and morning sputum were submitted to the National TB Reference Laboratory for culture. The culture result was needed for sensitivity analysis of the XPRES study and thus not included in this analysis. Initially and throughout the study, study nurses received training on how to collect a high quality sputum. Training included the importance of: (1) collecting a mucoid rather than salivary sample, (2) collecting the sample in a private but well-ventilated area outside the clinic, (3) use of a sputum collection job aid to guide patients through sputum production, and (4) use of observation and assistance (e.g. patting the back of the patient) to assist as they attempt to produce sputum. Guidelines were also provided on sample storage and refrigeration while awaiting transport by cooler box to the peripheral laboratory. At the beginning of the study, laboratorians at peripheral labs received refresher training on how to classify gross appearance of sputum. Training materials included a visual job aid, a coloured poster illustrating the different possible sputum appearance types and standard classification. Upon receiving a sample, a laboratorian assessed the macroscopic appearance and volume of sputum samples by visualization and recorded results. Sputum appearance was graded into categories of salivary, mucoid, muco-purulent and blood-tinged or other. Sputum volume was measured in millilitres using a pre-calibrated sputum collection bottle as a reference. After grading the appearance and volume of the sputum, the sputum sample was placed in a refrigerator until the time for smear or Xpert MTB/RIF testing.

### Data collection

Data were collected using standardized case report forms between August 2012 and November 2014. Data were double entered into a Clindex database (Fortress Medical Systems, Minneapolis, MN, USA). Inconsistencies and missing data were corrected through review of patient charts.

### Statistical analysis

Data were analysed using STATA (StataCorp. 2015. *Stata Statistical Software*: Release 14. College Station, TX: StataCorp LP) to fit univariate and multiple logistic regression models; to describe and compare demographic and clinical characteristics of patients; and to compare patients with specimen specified sputum characteristic vs. those without that characteristic. All analyses were adjusted for within-clinic correlation. We fit generalized-linear-mixed-models with appearance characteristics coded as salivary vs. non-salivary, mucoid vs. non-mucoid, and muco-purulent *vs* non-muco-purulent to estimate likelihood ratios (LR+) for sputum quality characteristics and the diagnostic yield of M. tuberculosis testing and 95% confidence intervals. These models were first used to construct omnibus tests for all appearance indicators and all volume indicators for Xpert MTB/RIF and smear. If the omnibus test was significant, we proceeded to interpret the LR+ and confidence interval. If the omnibus test was not significant, we considered all pairwise comparisons non-significant. The models included a random effect for clinic to adjust for within-clinic correlation. LR+s were defined as: 1) Prevalence of a given sputum characteristic in Xpert MTB/RIF MTB-positive samples divided by the prevalence of the same sputum characteristic in Xpert MTB/RIF MTB negative samples; 2) Prevalence of a given sputum characteristic in AFB smear positive of sputum samples divided by the prevalence of the same sputum characteristic in smear negative samples. P values of < 0.05 were considered statistically significant.

### Ethical considerations

The study protocol was approved by the Botswana Health Research and Development Committee, the Centers for Disease Control and Prevention, Atlanta, Georgia, Institutional Review Board (IRB), the University of Pennsylvania and the University of Witwatersrand, Johannesburg, South Africa IRB. Patients were enrolled following written informed consent process.

**Funding**: this research has been supported by the President’s Emergency Plan for AIDS Relief (PEPFAR) through the U.S. Centers for Disease Control and Prevention.

**Disclaimer**: the findings and conclusions in this report are those of the authors and do not necessarily represent the official views of the funding agency and the Centers for Disease Control and Prevention. References in this manuscript to any specific commercial products, process, service, manufacturer, or company does not constitute its endorsement or recommendation by the U.S. Government.

## Results

**Figure 1:**
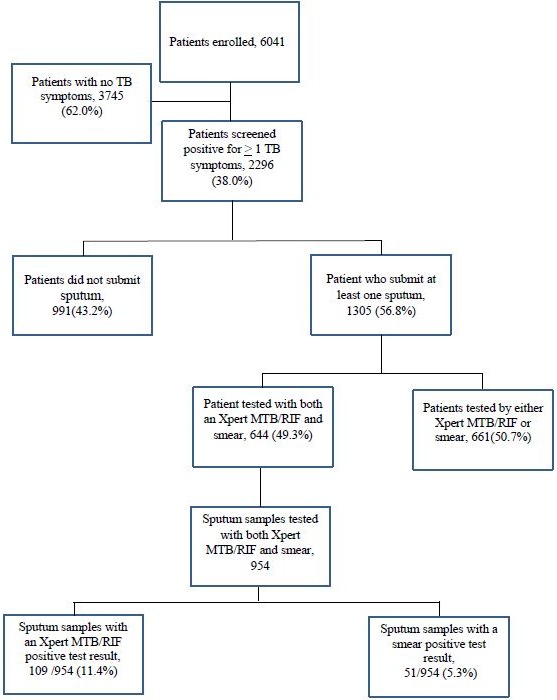
Patients enrolled, screened for tuberculosis symptoms and tested by Xpert MTB/RIF and smear

Among 6,041 patients prospectively enrolled 2,296 (38.0%) screened positive for TB symptoms at enrolment or follow-up. Of these, 1305 (56.8%) patients submitted > 1 sputum sample and 991 (43.2%) did not have an available sputum sample for testing; of the 1305, 644 (49.3%) patients had both smear and Xpert MTB/RIF test results and 661 (50.7%) were only tested by either Xpert MTB/RIF or smear and not both (Figure 1). Combined 991 and 661, the 1,652 patients will be referred to as patients that “did not receive sputum testing by both Xpert MTB/RIF and smear". Table 1 summarized the demographic and clinical characteristics of patients (1,652) that did not receive sputum testing by both Xpert MTB/RIF and smear and those patients (644) tested by both methods. Overall, the two populations were similar in terms of age, gender, CD4 status, history of previous TB treatment and presenting symptoms. Patients who did not receive sputum testing by both Xpert MTB/RIF and smear presented less frequently with cough (45.8% vs. 62.3%, aOR 0.50, p < 0.001) and smoking (24.3% vs. 30.9%, aOR 0.67, p = 0.008) than those tested by both smear and Xpert MTB/RIF (Table 1).

**Table 1:** Characteristics of patients with presumptive TB, by availability of sputum test results

	Did not receive sputum testing by both Xpert and smear			Tested by both Xpert and smear					
Characteristics	N	n	(%)	N	n	(%)	aOR*	95% CI**	*p value*
Age < 35	1652	723	43.8%	644	287	44.6%	0.93	0.76-1.14	0.460
Gender, female	1652	969	58.7%	644	368	57.1%	0.95	0.67-1.33	0.752
CD4 count < 200	1633	824	50.5%	636	317	49.8%	0.94	0.71-1.23	0.628
BMI < 18.5	1639	508	31.0%	644	171	26.6%	1.32	0.96-1.82	0.086
HgB < 10 mg/dl	1473	311	21.1%	564	116	20.6%	1.02	0.80-1.30	0.885
Previous TB	1649	237	14.4%	644	99	15.4%	0.90	0.66-1.23	0.487
Smoking***	1649	400	24.3%	644	199	30.9%	0.67	0.50-0.89	0.008
Alcohol use	1649	394	23.9%	644	170	26.4%	1.11	0.77-1.62	0.552
Miner	1649	114	6.9%	644	46	7.1%	0.97	0.66-1.41	0.861
**TB Symptoms**									
Cough	1652	756	45.8%	644	401	62.3%	0.50	0.36-0.70	<0.001
Fever	1652	380	23.0%	644	180	28.0%	0.83	0.65-1.08	0.154
Night-sweats	1652	392	23.7%	644	188	29.2%	0.86	0.64-1.17	0.326
Weight-loss	1652	955	57.8%	644	331	51.4%	1.44	1.00-2.09	0.052

* AOR adjusted Odds Ratio

** CI: Confidence interval.

*** Current or ex-smoker

### Predictive value of sputum appearance and volume on bacteriologically-confirmed diagnostic yield

From 644 patients, 954 sputum samples were tested by both Xpert MTB/RIF and smear; 43.3% (413) were salivary, 14.4% (137) were mucoid, 40.5% (386) were muco-purulent, and 1.0% (10) were blood tinged. The diagnostic yield of MTB detected by Xpert MTB/RIF was more than two-fold higher than that of smear (11.4% vs. 5.3%), p < 0.001). For any category of sputum quality measures (gross appearance or volume), the diagnostic yield of bacteriologically-confirmed diagnostic yield was approximately two-fold higher when tested by Xpert MTB/RIF compared to smear (Table 2). Sputum appearance was not predictive of bacteriologically-positive sputum by either Xpert MTB/RIF or smear (Omnibus test p > 0.05). The omnibus test for sputum volume for Xpert MTB/RIF testing was significant (p < 0.05) and sputum volume of 2ml to < 3ml was predictive of a positive sputum Xpert MTB/RIF result, LR+=1.26 (95% CI: 1.05 – 1.50). On the other hand, sputum volume 1ml to < 2ml was less predictive of a positive Xpert MTB/RIF result, LR+=0.62 (95% CI: 0.44-0.87). The proportion of bacteriologically-confirmed diagnostic yield showed no improvement by either Xpert MTB/RIF or smear as sputum quality improved (Table 2).

**Table 2:** Sputum characteristics from PLHIV with presumptive TB

Characteristics	n	Xpert MTB/RIF positive (%)	LR+ * (95% CI**)	Smear Positive (%)	LR+ (95% CI)
**Gross appearance^1^**					
Salivary	413	54 (13.1)	1.17 (1.00-1.37)#	27 (6.5)	1.24 (0.82-1.86)
Mucoid	137	14 (10.2)	0.88 (0.56-1.38)	5 (3.7)	0.67 (0.27-1.70)
Muco-purulent	386	38 (9.8)	0.85 (0.67-1.06)	19 (4.9)	0.92 (0.62-1.36)
Blood-tinged	10	0 (0.0)	n/a	0 (0.0)	n/a
Other	8	3 (37.5)	n/a	0 (0.0)	n/a
**Volume^2^**					
<1ml	2	0 (0.0)	n/a	0 (0.0)	n/a
1ml to < 2ml	272	20 (7.4)	0.62 (0.44-0.87)	7(2.3)	0.47 (0.22-1.01)
2 ml to < 3ml	330	46 (13.9)	1.26 (1.05-1.50)	25 (7.6)	1.45 (1.01-2.08)
3 ml to < 4ml	62	9 (14.5)	1.32 (0.65-2.68)	5 (8.1)	1.55 (0.75-3.20)
4 ml to < 5ml	28	2 (7.1)	0.60 (0.10-3.73)	0 (0.0)	n/a
>= 5ml	260	32 (12.3)	1.09 (0.72-1.64)	14 (5.4)	1.01 (0.62-1.63)
**Total**	954	109 (11.4%)^§^	-	51(5.3%)	-

* Likelihood ratio positive for bacteriologically-positive sputum.

** CI: Confidence interval.

# Lower confidence limit is rounded up from 0.995

§ Difference in proportions, bacteriologically-positive, between Xpert MTB/RIF and smear is significant. *p<0.001*

1 Omnibus tests for appearance (Xpert MTB/RIF and smear) not significant, *p>0.05.*

2 Omnibus test for volume was significant (*p<0.05*) for Xpert MTB/RIF, but was non-significant for smear

### Association between sputum appearance and TB diagnosis

**Figure 2:**
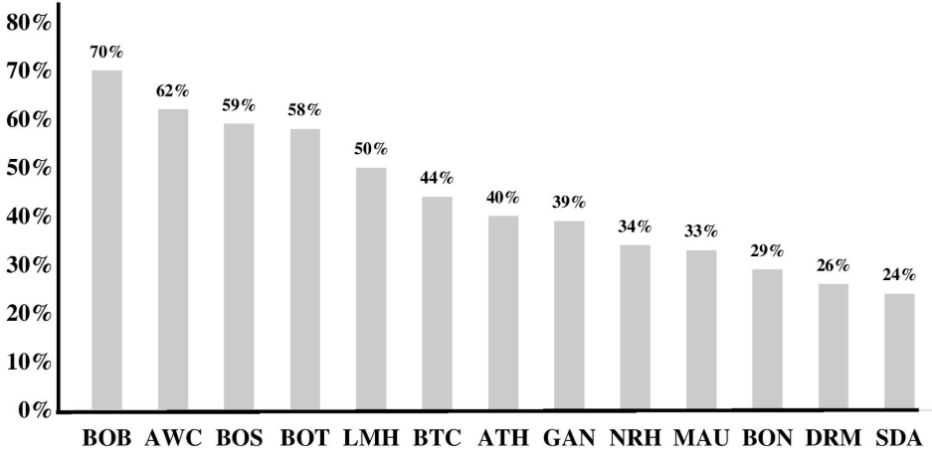
Proportion of salivary sputum by study sites

Of 954 tested sputum samples, 43.3% were classified as salivary. The percentage of patients with salivary sputum ranged from 24-70% (Figure 2; **Note**: Athlon Hospital = ATH, Area W Clinic = AWC, Bobonong Primary Hospital = BOB, Bontleng Clinic = BON, Boseja Clinic = BOS, Botswelelo Clinic = BOT, Broadhurst Traditional Clinic = BTC, Deborah Retief Primary Hospital = DRM, Gantsi Primary Hospital = GAN, Letsholathebe II Memorial Hospital = LMH, Maun Clinic = MAU, Molepolole Council Clinic = MCC, Nkoyaphiri Clinic = NKO, Nyangabgwe Referral Hospital = NRH Phuthadikobo Clinic = PHU, Seventh Day Adventist Hospital = SDA, Serowe Clinic = SER.) among study sites. Patient characteristics were similar among those who submitted salivary samples and non-salivary sputum samples (Table 3).

**Table 3:** Patient characteristics and quality of sputum

	Patients with salivary specimen			Patients with non-Salivary specimen					
Characteristics	N	n	(%)	N	n	(%)	aOR*	95% CI**	*p value*
Age < 35	315	133	42.2%	329	154	46.8%	0.82	0.56-1.22	0.314
Gender, female	315	183	58.1%	329	240	56.2%	1.08	0.79-1.51	0.649
CD4 count <200	311	148	47.6%	325	169	52.0%	0.80	0.61-1.04	0.094
BMI <18.5	313	90	28.8%	329	81	24.6%	1.31	0.83-2.01	0.228
HgB <10 mg/dl	285	60	21.1%	278	56	20.1%	1.04	0.61-1.77	0.893
Previous TB	315	44	14.0%	329	55	16.7%	0.73	0.46-1.15	0.162
Smoking***	315	94	29.8%	329	105	31.9%	0.83	0.55-1.27	0.367
Alcohol use	315	85	27.0%	329	85	25.8%	1.06	0.73-1.36	0.761
Miner	315	22	7.0%	329	24	7.3%	1.01	0.39-2.59	0.981
**TB Symptoms**									
Cough	315	192	61.0%	329	209	63.5%	0.81	0.42-1.57	0.506
Fever	315	86	27.3%	329	94	28.6%	0.96	0.53-1.73	0.876
Night-sweats	315	97	31.0%	329	91	27.7%	1.51	0.85-2.70	0.149
Weight-loss	315	163	51.8%	329	168	51.1%	0.99	0.65-1.52	0.976

* AOR adjusted Odds Ratio

** CI: Confidence interval

*** Current or ex-smoker

## Discussion

Our study demonstrated that among PLHIV with TB symptoms attending HIV care and treatment services, Xpert MTB/RIF was superior to smear at confirming the presence of MTB in sputum as previously reported [13-15]. Sputum gross appearance and quantity, however, were not predictive of bacteriologically-positive sputum by Xpert MTB/RIF or smear suggesting that despite a minimum required volume of sputum - especially for Xpert MTB/RIF testing - suboptimal quality of sputum might have affect the yield of bacteriologically-confirmed TB [16, 17]. These findings are consistent with a previous report by Ho *et al.* [18] who analysed over 20,000 sputum samples collected as part of an active case finding project in Vietnam; they reported that the macroscopic quality was similarly not predictive of bacteriologically- positive sputum. In the present study the only exception was sputum volume of 2ml to < 3ml that was predictive for a positive result when tested by Xpert MTB/RIF.

The percentage of samples classified as salivary in the present study was high at 43.3%, indicating potentially that, despite the training administered, a high proportion of patients were only able to produce salivary specimens. Recent studies from Kenya and Uganda also reported on the proportion of salivary sputa. In Kenya 44% of the samples were salivary and the study showed that salivary sputa had lower diagnostic yield than muco-purulent and mucoid sputa using Xpert MTB/RIF testing [10]. The study in Uganda examined presumptive TB patients screened with > 2 weeks of cough; the proportion of salivary sputa was at 16% [19]. In both the Kenya and the Uganda studies the proportion of salivary sample was not affected by HIV status. In Uganda, Xpert MTB/RIF test was conducted only among smear negative patients, and the diagnostic sensitivity, in contrast to our findings and those from Kenya, was significantly higher on salivary samples than mucoid sputa [19]. The Uganda findings on salivary sputum seems contrary to biological plausibility, showing higher diagnostic yield with lower quality sputa. However, the report from Uganda was consistent after comparison of diagnostic accuracy in reference to mycobacterial culture (higher culture positive among salivary than non-salivary). Given the higher positivity among salivary sputum samples was confirmed by culture, we are in agreement with Meyer et al that further study is essential exploring the possibility of potential dynamics affecting the Xpert MTB/RIF amplification in salivary sputum [19]. Ho *et al.* emphasized that assessment of sputum quality is a neglected aspect of accurate TB diagnostics [3]. Even though in the present study sputum quality was not predictive of diagnostic yield, we agree with Ho *et al.* that TB programs should continue to train providers on high quality sputum collection techniques. It is worth noting that despite the high cost of rolling out, Xpert MTB/RIF implementation in the world is expanding [1]. Such investments and their ultimate impact will potentially be compromised if TB diagnostic algorithms do not encompass collection of quality sputum [10]. A wide variability in the proportion of patients with salivary sample (24-70%) among clinics (Figure 2) suggest an inconsistency in sputum collection practices across the clinics. Bhat et al have shown an association of improved sputum quality and diagnostic yield [9]. It is time that TB screening and diagnostic algorithms include standardized methods of sputum collection and introduce a sputum collection system less prone to variability as suggested by Ho *et al.* [3].

In some previous reports, improving sputum quality increased TB diagnostic yield [18, 20-24]. Ho *et al.* were able to collect 99% mucoid or muco-purulent sputa by using sputum quality colour scale [18,20]; Alisjahbana *et al.*, used instruction to patients, where patients were individually addressed on the importance of sputum examination and how to produce adequate samples; this technique demonstrated an improvement in sputum volume collection with thicker consistency and resulting in higher smear positivity rates [21]; Sicsu *et al.* and Hirooka *et al.* using similar methods as Alisjahbana *et al.* showed higher quality and volume sputum samples that resulted in increased bacteriologically-confirmed TB diagnosis [22, 23]. Mhalu *et al.* demonstrated that sputum submission instructional videos increased the yield of TB cases through better quality of sputum samples [24]. In addition, training of health care workers and laboratorians on standardized methods of sputum collection and assessment of adequate good quality sputum can improve sputum quality [9] and measuring a volume in millilitres using a graded reference container can facilitate appropriate volume collection [9]. With these various methods, achieving improved quality and quantity of sputum were possible but well-designed studies are still needed to define a more comprehensive approach and standard.

While endeavouring to standardize sputum collection methods, under current clinical and laboratory practices, at least five reasons stand out about why salivary sputum should not be rejected: (1) a high percentage of salivary sputum are still being collected in clinical practice by health workers [10]; (2) sputum specimen appearance and volume are poor “negative predictors” of MTB in sputum [8, 18]; (3) a limited volume (only 1ml) of non-concentrated sputum maybe acceptable for Xpert MTB/RIF testing [6, 7]; (4) recent study demonstrate higher sensitivity of Xpert MTB/RIF in salivary samples than mucoid [19] and (5) above all, to minimize any missed opportunities for TB diagnosis [25]. Sputum rejection criteria, that consider salivary sample as unsuitable for testing should be reconsidered, particularly when using Xpert MTB/RIF testing [3]. In the present study, if salivary samples had been rejected, 12% and 6% of TB cases identified by Xpert MTB/RIF and smear, respectively, would have been missed as bacteriologically-confirmed TB case. Our study has limitations. Not all patients with TB symptoms were able to provide sputum, and for those who were able to provide sputum some did not receive sputum testing by both Xpert MTB/RIF and smear or were not tested at all. However, the patient characteristics among those tested by both Xpert MTB/RIF and smear and not tested by both or at all were similar. We also did not assess nor document laboratorian skills in assessing the quality of sputa. Furthermore, there may have been inter and/or intra-operator variability in assessing sputum quality [26].

## Conclusion

In conclusion, in the setting of HIV care and treatment, our study demonstrated that Xpert MTB/RIF was superior at confirming the presence of MTB in sputum samples from PLHIV at peripheral laboratories. However, sputum appearance and quantity were not consistently predictive for bacteriologically-confirmed positive sputum by Xpert MTB/RIF or smear. Despite a minimum required volume of sputum for Xpert MTB/RIF testing suboptimal quality of sputum have affected the yield of bacteriologically-confirmed TB. The high proportion of salivary samples warrants investigation and more effort should be made to re-train laboratorians and clinical staff in collecting good quality sputum and classifying sputum appearance in a standard manner.

### What is known about this topic

- As with smear, sputum quality may predict the likelihood of obtaining a bacteriologically-confirmed TB when using Xpert MTB/RIF;
- Xpert MTB/RIF was superior to smear at confirming the presence of *Mycobacterium tuberculosis* in sputum.

### What this study adds

- Due to poor quality (high proportion of salivary samples) sputum appearance and quantity were not consistently 0predictive for bacteriologically-confirmed positive sputum by Xpert MTB/RIF or smear;
- The need for re-train laboratorians and clinical staff in collecting good quality sputum and classifying sputum appearance in a standard manner;
- Despite a minimum required volume of sputum for Xpert MTB/RIF testing suboptimal quality of sputum might have affected the yield of bacteriologically-confirmed TB.

## Competing interests

The authors declare no competing interests.
